# Pharmacodynamics of TRPV1 Agonists in a Bioassay Using Human PC-3 Cells

**DOI:** 10.1155/2014/184526

**Published:** 2014-02-02

**Authors:** Daniel Alvarez-Berdugo, Marcel Jiménez, Pere Clavé, Laia Rofes

**Affiliations:** ^1^Laboratori de Fisiologia Digestiva, Departament de Cirurgia, Hospital de Mataró, Universitat Autònoma de Barcelona, 08304 Mataró, Spain; ^2^Centro de Investigación Biomédica en Red de Enfermedades Hepáticas y Digestivas, 28029 Madrid, Spain; ^3^Departament de Biologia Cel*·*lular, Fisiologia i Immunologia, Universitat Autònoma de Barcelona (UAB), 08193 Bellaterra, Spain

## Abstract

*Purpose*. TRPV1 is a multimodal channel mainly expressed in sensory neurons. We aimed to explore the pharmacodynamics of the TRPV1 agonists, capsaicin, natural capsaicinoids, and piperine in an *in vitro* bioassay using human PC-3 cells and to examine desensitization and the effect of the specific antagonist SB366791. *Methods*. PC-3 cells expressing TRPV1 were incubated with Fluo-4. Fluorescence emission changes following exposition to agonists with and without preincubation with antagonists were assessed and referred to maximal fluorescence following the addition of ionomycin. Concentration-response curves were fitted to the Hill equation. *Results*. Capsaicin and piperine had similar pharmacodynamics (*E*_max_ 204.8 ± 184.3% piperine versus 176.6 ± 35.83% capsaicin, *P* = 0.8814, Hill coefficient 0.70 ± 0.50 piperine versus 1.59 ± 0.86 capsaicin, *P* = 0.3752). In contrast, capsaicinoids had lower *E*_max_ (40.99 ± 6.14% capsaicinoids versus 176.6 ± 35.83% capsaicin, *P* < 0.001). All the TRPV1 agonists showed significant desensitization after the second exposition and their effects were strongly inhibited by SB366791. *Conclusion*. TRPV1 receptor is successfully stimulated by capsaicin, piperine, and natural capsaicinoids. These agonists present desensitization and their effect is significantly reduced by a TRPV1-specific antagonist. In addition, PC-3 cell bioassays proved useful in the study of TRPV1 pharmacodynamics.

## 1. Introduction

The transient receptor potential family V member 1 cation channel (TRPV1) is a nonselective channel that responds to noxious stimuli such as low pH, painful heat, and irritants. Identification of TRPV1 through molecular cloning such as the capsaicin receptor [1] represented a milestone in the study of noxious stimuli. Since then, many studies have tried to determine its druggability and pharmacological characterization [2–4].

TRPV1 is mainly expressed in unmyelinated C fibers and in A*δ* thinly myelinated fibers of the dorsal root (DRG), trigeminal ganglion (TG), and visceral afferent fibers and it plays an important role in temperature and pain perception. However, TRPV1 expression is not exclusive to neuronal tissue but can be found in other locations such as urothelial and epithelial cells [5, 6], which respond first to irritating and inflammatory stimuli together with afferent nociceptors [7, 8].

Upregulation of TRPV1 expression has been observed in various diseases [9] including those associated with visceral hypersensitivity such as irritable bowel syndrome [10] and other diseases like chronic cough [11] and neuropathic pain [12]. Downregulation of TRPV1 has been observed in some diseases such as periodontitis [13]. Strategies aiming to block or desensitize TRPV1 have been explored to treat several diseases such as neuropathic pain, spinal detrusor hyperreflexia, bladder hypersensitivity, and pruritus [3]. Hence, TRPV1 agonists and antagonists have become essential pharmacological tools to address the treatment of these disorders.

Most bioassays aiming to characterize the pharmacodynamics of these TRPV1 ligands use human cells such as HEK293 heterologously expressing TRPV1 [14]. However, this technology is not available for most research groups and has some limitations in studies concerning excitable receptors due to the differences between the original cell and the engineered one [15]. There are also problems derived from the heterologous expression technique such as insertional alterations. Recently, Sánchez et al. [16] demonstrated the natural expression of TRPV1 in two human prostate cell cultures: PC-3 and LNCaP. They did this by means of retrotranscription polymerase chain reaction (RT-PCR), Western Blot, and binding studies. Thus, PC-3 was shown to be a human prostate epithelial cancer cell line that could be used to examine TRPV family receptors pharmacodynamics in future studies.

The aim of the present study was to explore the pharmacodynamics of TRPV1 agonists (capsaicin, natural capsaicinoids, and piperine) in a bioassay using human PC-3 cells prior to performing a clinical study.

## 2. Material and Methods

### 2.1. Experimental Design

In this bioassay, we compared the pharmacodynamics of capsaicin, natural capsaicinoids, and piperine and studied the effect of desensitization after repeated exposure and the effect of the specific TRPV1 antagonist, SB366791.

### 2.2. Cell Culture and Calcium Imaging

Human Caucasian prostate adenocarcinoma PC-3 cells expressing TRPV1 (ECACC Catalogue no. 90112714, Salisbury, UK) were grown in RPMI 1640 medium supplemented with 10% fetal bovine serum, 100 UI/mL penicillin G, 100 *μ*g/mL streptomycin, and 0.25 *μ*g/mL amphotericin B (all from Invitrogen, Paisley, UK) at 37°C and 5% CO_2_. Cells were passed every 3-4 days and only cells from passage 25 or lower were used in the study. Twenty-four hours before the experiment, cells were passed to a 35 mm plate with a glass cover slip. Before the assay, cells were incubated with extracellular medium (140 mM NaCl, 4.8 mM KCl, 1 mM MgCl_2_·6H_2_O, 1.8 mM CaCl_2_·2H_2_O, 10 mM glucose, and 10 mM HEPES; pH 7) containing 3.64 *μ*M of Fluo-4 AM (Molecular Probes, Eugene, OR, USA) at room temperature for 45 min. Cover slips were then mounted in an imaging chamber and continuously perfused with the extracellular medium. The cells were imaged with IX-FLA equipment (Olympus Biosystems, Heidelberg, Germany) connected to an Olympus IX70 microscope with a 20x lens. The timing of the experiments consisted of 10 s agonist expositions, 5 min antagonist incubations, and 10 min of interexposition periods. Ionomycin (10 *μ*M) was added at the end of each experiment as a control of fluorescence. Images were recorded for 40 s at 2.5 Hz, and fluorescence emission changes were measured after each agonist addition at 488 nm.

### 2.3. Drugs and Reagents

Capsaicin, SB366791 (both from Tocris, Bristol, UK), piperine, and ionomycin (from Sigma Aldrich, St Louis, MO, USA) were dissolved in dimethyl sulfoxide (DMSO) 1 mM. Final concentrations were obtained by dissolution of drugs in extracellular medium. Capsaicinoid concentration in capsaicinoid sauce (McIlhenny Co, Avery Island, LA, USA) was determined using liquid chromatography (AOAC 995.03) and was 185.5 *μ*g/g, the final desired concentration being obtained by dissolution in extracellular medium.

### 2.4. Data Analysis

**Figure 1:**
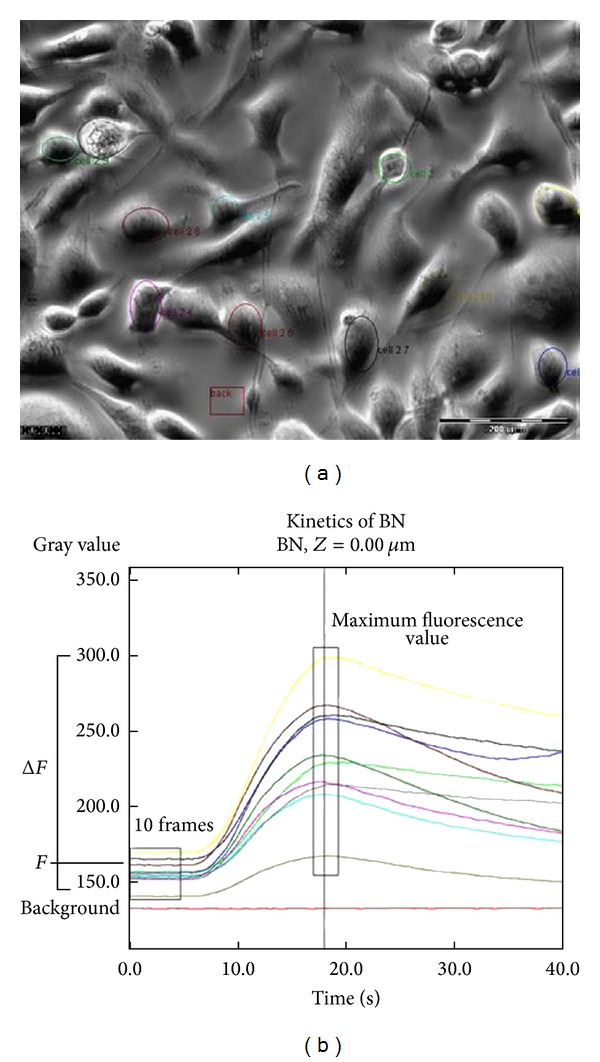
Calcium imaging data analysis. The regions of interest (ROIs) of 10 cells per plate (colored ellipsis) are marked to measure changes in fluorescence and a ROI without cells (dark red rectangle) is also marked to measure the background signal (a). Increases in fluorescence are plotted as a time function for each cell. The increase of fluorescence (Δ*F*) is calculated as the difference between the mean of the peak and 2 frames backward and forward (narrow rectangle measurements) and the mean of the measurements of 10 frames before agonist exposition (wide rectangle of measurements); the background signal (red line) is then subtracted (b).

The analysis was performed with the Cell software (Olympus Biosystems, Heidelberg, Germany). Fluorescence from individual cells was monitored as a function of time by drawing a region surrounding the inner part of the cell membrane, the region of interest (ROI), and measuring the total fluorescent signal from this region. A total of 10 ROI were selected from each plate. Maximum intensity of fluorescence (*F*_max⁡_) was measured at the end of the experiment following the application of the Ca^2+^ ionophore, ionomycin (1 *μ*M). Background fluorescence was determined by measuring the fluorescent signal of a ROI without a cell and was subtracted from the increases in cell fluorescence (Δ*F*). These were calculated as the mean of the peak fluorescence value and the fluorescence value of 2 frames before and 2 frames after the peak, minus the basal fluorescence at the onset of the recording (the mean fluorescence value over the 10 first frames) (Figure 1). All experiments were carried out at room temperature (18–20°C).

### 2.5. Statistical Methods

Concentration-response curves were fitted to the Hill equation. Variables in the equation are shown as mean ± SEM and contrasted by means of a Student *t*-test for nonpaired data. The nonparametric Mann-Whitney *U* test was used to analyze desensitization and antagonism. Statistical significance was accepted if *P* values were less than 0.05. Statistical analysis was performed using GraphPad Prism 5.01 (San Diego, CA, USA).

## 3. Results

### 3.1. Pharmacodynamics of TRPV1 Agonists

**Figure 2:**
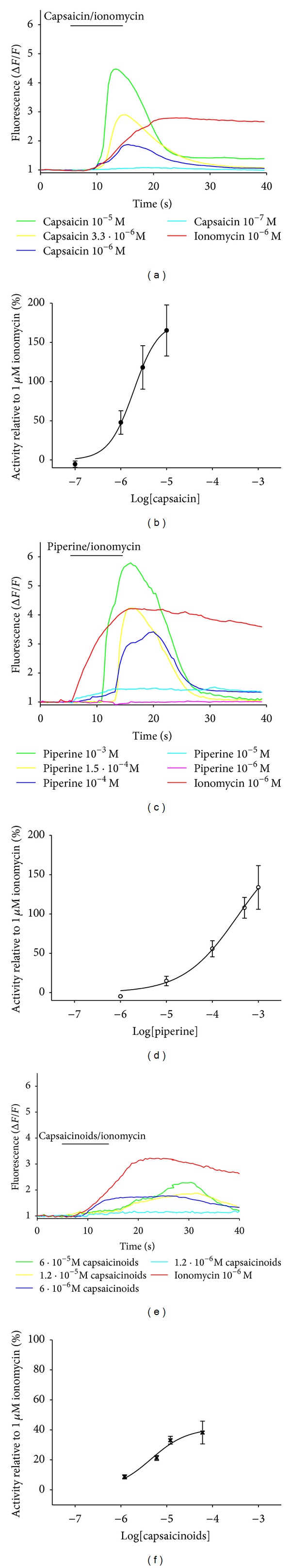
Calcium transients and dose-response curves. The means of measurements for each agonist concentration are plotted as time functions of the increase in fluorescence related to the signal before exposition (a, c, and e); ionomycin was applied at the end of each experiment and its maximum effect was used to normalize the effect of the different agonists and concentrations. The slope showing calcium entrance induced by capsaicin and piperine exposition (capsaicin 830.1 ± 128.1 relative increase of fluorescence per second and piperine 1238 ± 146.8 relative increase of fluorescence per second) (a, c) is sharper than the slope showing calcium entrance induced by natural capsaicinoids exposition (184.6 ± 17.39 relative increase of fluorescence per second) (e). The normalized effect of each concentration is plotted as a dose-response curve for each agonist. Each point represents the mean ± SEM of 3 independent experiments (*n* = 10) (b, d, and f).

Capsaicin, the reference agonist of TRPV1, and piperine caused a concentration-dependent response in PC-3 cells, achieving maximal effect at 10^−5^ and 10^−3^ M, respectively, with similar *E*_max⁡_ (204.8 ± 184.3% piperine versus 176.6 ± 35.83% capsaicin, *P* = 0.8814) and Hill coefficient (0.70 ± 0.50 piperine versus 1.59 ± 0.86 capsaicin, *P* = 0.3752) and not significantly different EC_50_ (4.14 · 10^−4^ M piperine versus 1.90 · 10^−6^ M capsaicin, *P* = 0.0675). Natural capsaicinoids also caused a concentration-dependent intracellular calcium increase, with significantly lower *E*_max⁡_ than capsaicin (40.99 ± 6.14% capsaicinoids versus 176.6  ±  35.83% capsaicin, *P* < 0.001), and similar EC_50_ (4.36 · 10^−6^ M capsaicinoids versus 1.90 · 10^−6^ M capsaicin, *P* = 0.1601) and Hill coefficient (1.18 ± 0.45 capsaicinoids versus 1.59 ± 0.86 capsaicin, *P* = 0.674) (Figure 2).

### 3.2. Specificity: Desensitization and the Effect of an Antagonist

**Figure 3:**
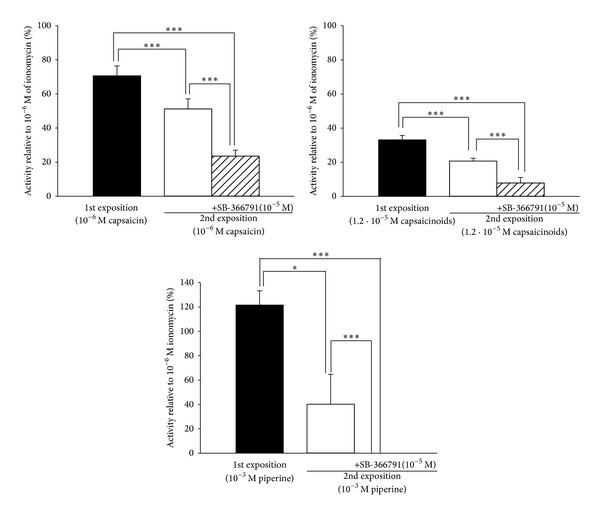
Desensitization and antagonist effect. Capsaicin, capsaicinoids, and piperine normalized first exposition effect (black box) is compared with their normalized second exposition effect (white box) and SB-366791 incubation before second exposition effect (striped box). Data is expressed as mean ± SEM. *P < 0.05, ***P < 0.001.

Repetitive expositions to capsaicin (10^−6^ M, 10 min interexposition), piperine (10^−3^ M and 10^−4^ M, 10 min interexposition), or natural capsaicinoids 1.2 · 10^−5^ M (10 min interexposition) significantly reduced the response of PC-3 cells. The effect of capsaicin was reduced by 38.31 ± 4.08%, piperine by 67.61 ± 5.31%, and capsaicinoids by 22.30 ± 2.24% after second exposition (Figure 3). In addition, 5 min incubation with the specific TRPV1 antagonist SB366791 (10^−5^ M), strongly antagonized the response of capsaicin (10^−6^ M), piperine (10^−3^ M), and capsaicinoids (1.2 · 10^−5^ M). Following 5 min incubation with the antagonist and 5 min wash before second exposition, the effect of capsaicin was reduced by 74.66 ± 2.93%; piperine by 100 ± 0.005%, and capsaicinoids by 91.71 ± 2.76% (Figure 3).

## 4. Discussion

The results show that capsaicin and piperine to a greater degree and natural capsaicinoids to a lesser degree successfully stimulate the TRPV1 channel. Repeated exposition of these agonists decreases the effect on TRPV1, suggesting desensitization. Moreover, their effect is significantly reduced by a TRPV1-specific antagonist, showing their action to be specific to this receptor. PC-3 cells were found to be perfectly adequate tools to study TRPV1 pharmacodynamics.

Caterina et al. studied the pharmacology of rVR1 when they first cloned it [1]. Since then, several groups have evaluated the use of TRPV1 as a therapeutic target to treat various diseases [3, 4]. In our research, TRPV1 is a promising target to treat oropharyngeal dysphagia, a major complaint among the elderly and patients with neurological diseases, and one characterized by pharyngeal and laryngeal sensory deficits and delayed and prolonged swallow response [17]. Previous clinical studies showed capsaicin and piperine to be effective in improving the swallowing response [18, 19] but we needed more knowledge of the TRPV1 agonists' pharmacodynamics to design a clinical trial to assess proof of concept [20]. We used a bioassay to evaluate the pharmacology of capsaicin, piperine, and natural capsaicinoids on human TRPV1 constitutively expressed in PC-3 cells.

In our study, capsaicin and piperine had similar *E*_max⁡_ and Hill coefficient values and we did not find significant differences in their EC_50_ values, while comparable assays found a lower maximum effect for piperine and significant differences in their EC_50_ [21, 22]. We also found that natural capsaicinoid sauce has lower *E*_max⁡_ than capsaicin or piperine. This could be explained by the fact that capsaicinoid sauce contains different capsaicinoids, one of which is capsaicin, found in previous studies to have the greatest pungent effect among vanilloids [23]. Our results helped us determine the following optimal concentrations for the clinical trial: 10 *μ*M for capsaicin, 150 *μ*M to 1 mM for piperine, and 150 *μ*M for capsaicinoid sauce [20].

Our results also showed that all the agonists tested undergo desensitization after repeated exposition. Capsaicin had already been shown to desensitize TRPV1 action [1, 21, 24], and Liu and Simon had shown that piperine also desensitizes TRPV1 action [21], but in our assay piperine desensitized TRPV1 action to a greater degree.

Finally, the use of TRPV1-specific antagonist SB-366791 allowed us to verify that the effect of our agonists is specific to TRPV1. In previous studies, capsazepine was used as a specific vanilloid receptor antagonist to assess agonist specificity, but it has nonselective actions on other receptors and apparent modality-specific properties. SB-366791, however, is a TRPV1 antagonist with high potency and improved selectivity profile with respect to other commonly used TRPV1 antagonists [25]. Another difference between our study and previous ones is the assay design with PC-3 cells, a system that has been rarely used in comparison with transfected HEK293 cells. This study shows that PC-3 cells are a good surrogate to test TRPV1 pharmacology and more accessible as they do not require transfection.

## 5. Conclusion

In summary, we have examined the pharmacological parameters of TRPV1 agonists and analysed desensitization and the effect of the antagonist SB366791 using an alternative methodology more easily accessible to most research groups. We reached our goal of testing capsaicin, piperine, and natural capsaicinoid pharmacodynamics for subsequent clinical assays.
